# A Cross-Level Study on Family Involvement and Job Satisfaction

**DOI:** 10.3389/fpsyg.2018.01547

**Published:** 2018-08-28

**Authors:** Hongguo Wei, Jinqiang Zhu, Hai Li, Diana Bilimoria

**Affiliations:** ^1^University of Central Oklahoma, Edmond, OK, United States; ^2^Minzu University of China, Beijing, China; ^3^Beijing Normal University, Beijing, China; ^4^Case Western Reserve University, Cleveland, OH, United States

**Keywords:** family involvement, job satisfaction, (in)congruence, cross-level, working couples

## Abstract

This study aims to explore how various dynamics of working couples’ family involvement shape their job satisfaction. With a sample collected from primary school teachers and spouses in China (*n* = 236), we use polynomial regression, response surface method, and multilevel structural equation model to capture the various dynamics of working couples’ family involvement. We found that (1) high-high spouses’ family involvement has a negative impact on individual job satisfaction, and low-low spouses’ family involvement is positively related to individual job satisfaction. (2) High-high spouses’ family involvement benefits the creation of positive affect at the family level, which decreases family-to-work conflict and mitigates its negative impacts on individual job satisfaction. (3) Working couple’s perceived work-to-family enhancement moderates the relationship between spouses’ family involvement and positive affect at the family level. This study extends our understanding of family-to-work spillover effects from the viewpoint of dynamic interaction between spouses at the cross level.

## Introduction

Role involvement, defined as “ego or psychological involvement" and “a preoccupation" with one domain that makes a person “unavailable to perform the demands or responsibilities of the other domain" (Aryee et al., 2005: 135), is an important variable impacting work-family interface. There have been inconsistent findings regarding the impact of one’s family involvement on work-related outcomes. Some scholars assert that an individual’s high involvement in the family domain initiates family-work conflict and thus constrains his or her job performance and job satisfaction (e.g., Frone et al., 1992; Aryee et al., 1999; McMillan et al., 2011). Some others posit that family involvement may decrease family-work conflict in the view of social support (Adams et al., 1996). Adams et al. (1996) found that an individual’s high family involvement was related to a high level of family social support (emotional but not instrumental), which further decreased the family interfering with work. We assume that further examinations of the level of analysis, different mechanisms, and contextual variables enrich our understanding of this inconsistency. Yet there is a paucity of research examining the whole interface between the working couples. There is even less research examining how various dynamics of a working couple’s family involvement impact job satisfaction by looking at the underlying mechanism and contextual variables affecting this relationship (Allen and Martin, 2017).

In the present study we draw on the resource accumulation perspective in the expansion model of personal resources (Greenhaus and Parasuraman, 1999; Rothbard and Edwards, 2003; Greenhaus and Powell, 2006; Gordon et al., 2007) to explore how various dynamics of working couples’ family involvement (e.g., high-high, high-low, and low-low) influences their respective job satisfaction. The resource accumulation perspective assumes that one’s resources are abundant and expandable, and that undertaking multiple roles brings privileges, status security, social capital and other resources, and self-esteem (Sieber, 1974). The accumulated resources from undertaking additional roles spill over from one domain to the other domain for one’s own benefit (Greenhaus and Powell, 2006), and thus the spillover from one domain to another can be positive (i.e., an enrichment process) (Marks, 1977; Kirchmeyer, 1992; Rothbard, 2001; Gordon et al., 2007). Rather than simply embracing the viewpoint that the resources invested in one domain necessitates sacrifices in the other (i.e., a depletion process) as a result of distinct norms and demands in work and family domains (Rothbard, 2001; Aryee et al., 2005), we argue that the dynamics occurring between the spouses at the whole interface may reduce the negative impact of family involvement on job satisfaction. We further posit positive affect at the family level and tensions between work and family domains (i.e., family-work conflict) as two mediators, and the working couple’s perceived work-to-family enhancement as a moderator explaining this dynamic relationship.

This study contributes to work-family research from the following aspects. First, as a response to the call for work-family research at family and work group levels (e.g., Frone, 2003; Eby et al., 2005; Liu et al., 2016), we examine the various dynamic combinations of working couples’ family involvement. Despite scholarly effort to examine positive spillover from family to work domain for individuals (e.g., Ten Brummelhuis et al., 2014), very limited, if any, research has taken into account of how the spillover at the marital dyad level impacts the relationship between family involvement and job satisfaction for spouses who are both employed. Second, we advance existing research by explaining the inconsistent findings of one’s family involvement and job satisfaction. This study shows that taking into account of the cross-level analysis, mediators, and contextual factors enrich our understanding of the inconsistency. Third, we uncover the underlying mechanisms mitigating the negative impact of family involvement on job satisfaction. An examination at the whole interface of the working couples allows us to investigate the mediation of positive affect at the family level and family-work conflict, which have been overlooked in extant research.

## Theoretical Background and Hypotheses

### Spouses’ Family Involvement and Job Satisfaction

Family involvement signifies psychological involvement and preoccupation in the family domain, and thus less availability for the demands of the work domain (Greenhaus and Parasuraman, 1999; Aryee et al., 2005; Du et al., 2018). When spouses both highly value involvement in family life (that is, high-high spouses’ family involvement), it benefits the family’s well-being. Yet in this case, they are likely to attend to problems related to family role demands, which increases the possibility of family interfering with the requirements of their respective work roles (Rothbard, 2001). Evidence has linked family involvement to increased family-work conflict (Frone et al., 1992; Adams et al., 1996) and thus decreased job satisfaction (Adams et al., 1996). The high-high spouses’ family involvement means that they both value the importance of being highly involved in family life and thus will have relatively little time and energy on work roles, which buffers their individual work outcome and experience of job satisfaction. Yet when spouses are both involved in family life at a low level (that is, low-low spouses’ family involvement), they would both be more likely to spend more time, energy, and resources in the work domain leading to the experience of higher job satisfaction.

Hypothesis 1: High-high spouses’ family involvement is negatively related with job satisfaction, while low-low spouses’ family involvement is positively related with job satisfaction.

### Spouses’ Family Involvement and Positive Affect at the Family Level

According to resource accumulation perspective, an individual’s involvement in one domain (role A) generated resources that benefit another domain (role B) through the high performance and positive affect created in role A (Greenhaus and Powell, 2006). An individual who is highly involved in family life may generate relevant resources of different types (e.g., skills, perspectives, psychological and physical resources, and social capital resources), which are linked to positive affect in the family role. When looking at the working couples, we define positive affect at the family level as a supportive emotional environment that a couple mutually creates, which is operationalized as the shared positive affect between them. Building on Rothbard (2001) finding of a positive relationship between family engagement and individual positive affect, we argue a positive association between family engagement and positive affect at the family level.

Specifically, when both spouses highly value family involvement (i.e., high-high), they would be more likely to understand and support each other in the family domain and thus develop positive affect at the family level. When the spouses both have a low value of family involvement (i.e., low-low), they both would likely prefer to sacrifice family time to undertake work requirements, and this decreases the possibility of creating positive affect at the family level. Further, when there is an incongruence of spouses’ family involvement, it means one is highly involved in family life while the other is less involved in family life (i.e., high-low and low-high). This disequilibrium between the couple is likely to challenge either spouse’s contribution to the family and thus cause a negative impact on positive affect at the family level. Thus, we hypothesize that:

Hypothesis 2a: High-high spouses’ family involvement is positively related with positive affect at the family level, while low-low spouses’ family involvement is negatively related with positive affect at the family level.
Hypothesis 2b: The incongruence of spouses’ family involvement (e.g., high-low and low-high) is negatively related to positive affect at the family level.

### Mediation of Family-Work Conflict

Building on resource accumulation, there is positive spillover and enrichment from one domain to the other, such that the support one has in a family relationship may have a positive impact on their work and vice versa (Crouter, 1984; Kirchmeyer, 1992; Michel et al., 2011). In this regard, the positive spillover from family (such as positive affect at the family level) may reduce or counterbalance the negative impact between family and work roles (Crouter, 1984), thus reducing family-work conflict. Family-work conflict refers to interference from the family domain to the work domain (Gutek et al., 1991; Frone et al., 1992). We thus propose that positive affect at the family level is a potential resource to reduce strain-based family-work conflict (see Michel et al., 2011). In addition, research indicates that family-work conflict leads to negative work-related outcomes, such as decreased job satisfaction (Netemeyer et al., 1996; Kossek and Ozeki, 1998; Frone, 2003; Gordon et al., 2007; Amstad et al., 2011). As such, we propose that family-work conflict mediates the relationship between positive affect at the family level and job satisfaction.

Hypothesis 3a: Family-work conflict mediates the relationship between positive affect at the family level and job satisfaction.

Integrating hypotheses 2a, 2b, and 3a, we propose that spouses’ family involvement impacts job satisfaction through positive affect at the family level and family-work conflict.

Hypothesis 3b: Positive affect at the family level and family-work conflict mediate the relationship between the (in)congruence of spouses’ family involvement and job satisfaction.

### Moderated Effect of Working Couple’s Perceived Work-to-Family Enhancement

Based on the resource accumulation perspective, the positive affect and experience from work spill to the family domain and enhance the family life (Greenhaus and Parasuraman, 1999; Gordon et al., 2007). We define a working couple’s perceived work-to-family enhancement as the couple’s shared perception regarding how their work enriches family life.

High working couple’s perceived work-to-family enhancement means positive spillover from the work to the family domain, which benefits the spouses’ family life through positive affect, developmental resources, and psychosocial capital from the work domain (Carlson et al., 2006). A meta-analysis of 21 studies showed that work-to-family enhancement is positively related to one’s satisfaction with family life (McNall et al., 2010). Thus high working couple’s perceived work-to-family enhancement may strengthen the positive impact of high-high spouses’ family involvement on positive affect at the family level. In addition, when spouses both have a low level of family involvement, positive spillover from the work domain (such as a sense of success and meaningfulness) may increase the quality of their family life. Thus, when there is a high level of working couple’s perceived work-to-family enhancement, the negative impact of low-low spouses’ family involvement on positive affect at the family level may be mitigated. Further, the positive spillover from work to family may reduce the negative impact of incongruent family involvement on positive affect at the family level such that the detrimental effect derived from the inconsistent family value may be weakened.

Hypothesis 4: The higher the level of working couple’s perceived work-to-family enhancement, (a) the stronger the positive relationship between high-high spouses’ family involvement and positive affect at the family level, and the weaker the negative relationship between low-low spouses’ family involvement and positive affect at the family level; (b) the weaker the negative relationship between the incongruence of spouses’ family involvement and positive affect at the family level.

**FIGURE 1:**
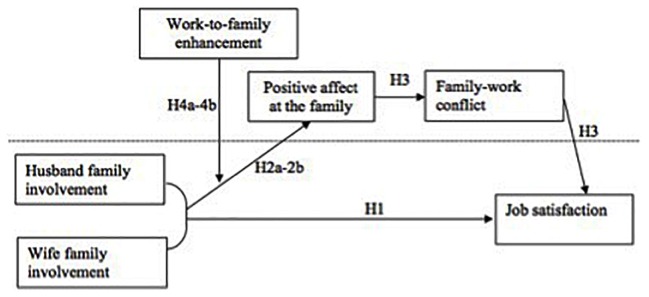
Theoretical framework of spouses’ family involvement and job satisfaction.

Our overall theoretical framework is presented in **Figure 1**.

## Method

### Sample and Data Collection

We recruited working couples from primary public school teachers and spouses in northern China. All of them were opposite-sex couples. We received the approval of each school principle before recruiting participants. We contacted each school coordinator for the names and code numbers of all the teachers and then selected participants by systematic sampling (i.e., randomly selected the first participant and then selected another one with a fixed interval). We also invited their spouses to participate. We gave each teacher a sealed envelope with two informed consent forms and two questionnaires. Participation in the study was entirely voluntary. If participants did not want to participate in the study, they were asked to return the sealed envelope without signing the informed consent form. The two questionnaires were marked with different colors, respectively, for the teacher and the spouse. All responses were anonymous to protect the privacy of participants. The teacher and the spouse were asked to independently fill in their questionnaire and return them to the school coordinator in a prepared sealed envelope. We compensated each couple with one hundred Chinese yuan (about 15.45 USD) for their participation and time. We sent out 300 pairs of questionnaires and got back 281 pairs of matched data. We achieved the high response rate (93.67%) because of the school official’s support and the compensation.

Given the research purpose, we then deleted 45 couples with missing values in gender, one spouse unemployed, or inappropriate to be aggregated to the dyad level. The valid sample was 236 in the end and the valid response rate was 78.67%, with 89% of the teachers being female. Of the 236 couples, 85.20% had children. The average age of the wife and husband was 38.63 years old (SD = 6.61) and 40.82 years old (SD = 6.93). Seventy-eight percent of the husbands were middle and low-level managers or associates in a variety of enterprises. Eighty-nine percent of wives were teachers, and 8% were middle and low-level managers in enterprises. The average work time was 45.67 h for wives and 46.81 h for husband, far beyond their respective time spent on housework (15.90 h for wives, and 13.61 h for husbands). The average job tenure was 12.50 years (SD = 8.61) for wives and 14.67 years (SD = 9.41) for husbands.

### Measures

We followed the translation and back-translation process for all the measures from English to Chinese (Brislin, 1970). We used a 6-point Likert scale anchored by 1 = completely disagree and 6 = completely agree for all the measures.

#### Family Involvement

Family involvement was measured with four items adapted from the job involvement scale by Kanungo (1982) to fit the family context. Sample item includes “Family should be considered central to life” (α = 0.87 for wives, and 0.86 for husbands).

#### Positive Affect at the Family Level

We used Price (2001) 4-item positive affect scale to measure individual positive affect, and aggregated it to measure positive affect at the family level. Sample item includes “Most days I have moments of real fun” (α = 0.86 for wives, and 0.87 for husbands).

#### Family-Work Conflict

We measured family-work conflict using four items from Netemeyer et al. (1996). Sample item includes “I have to put off doing things at work because of demands on my time at home” (α = 0.89 for wives, and 0.93 for husbands).

#### Working Couple’s Perceived Work-to-Family Enhancement

Working Couple’s Perceived work-to-family enhancement was measured using four items from Lu et al. (2009) work-family facilitation scale, which was revised based on Grzywacz and Marks’ (2000) work-family spillover scale. Sample item includes “The things I do at work make me a more interesting person at home” (α = 0.87 for wives, and 0.86 for husbands).

#### Job Satisfaction

Job satisfaction was measured with three items from Brayfield and Rothe (1951) job satisfaction index which was revised by Aryee et al. (1999). Sample item includes “I feel fairly well satisfied with my present job” (α = 0.83 for wives, and 0.82 for husbands).

#### Control Variables

We controlled for the number of children, age, job tenure, work time, and family time. Extant studies with Chinese or non-Chinese samples confirm that these variables affect work-family conflicts or job satisfaction (e.g., Frone et al., 1992; Isenhour et al., 2012; Li et al., 2013).

### Analytical Strategy

We employed *SPSS* 22.0 and *Mplus* 7.0 for data analyses. Prior to testing the hypotheses, we examined whether the variables of interest were appropriate to be aggregated to the family level. Following LeBreton and Senter (2007), we deleted paired cases with *R_wg_* (i.e., within-group interrater reliability) smaller than 0.30 for all the variables with a proposed meaning at the family level. *R_wg_* was used to test the extent to which judges “agreed” on a set of judgments (James et al., 1984). After cleaning the sample, we differentiated the matched-data with the roles of being a husband or a wife. We then transformed the data format from dyads to pairwise in order to do the cross-level analysis.

Our overall data analysis plan was as follows: First, we examined the possibility of common method variance (CMV) with Harman’s single-factor test and unmeasured latent methods factor (Podsakoff et al., 2003). Second, we examined the discriminant validity of the variables of interest in this study with confirmatory factor analysis (CFA) using *Mplus*. Then, we adopted cross-level polynomial regression and surface response modeling (Edwards and Parry, 1993; Jansen and Kristof-Brown, 2005) to test hypotheses 1, 2, and 4 using *Mplus*. We used maximum likelihood (ML) for model estimation. Five polynomial terms were calculated with the pooled grand mean centered data in order to reduce multicollinearity. Further, we tested the mediating effect of family-work conflict on positive affect at the family level and job satisfaction (hypothesis 3) and the mediating effect of positive affect at the family level and family-work conflict on family involvement and job satisfaction (hypothesis 3a) with cross-level structural equation modeling (SEM) using *Mplus* by treating the five polynomial terms as a block variable (Edwards and Cable, 2009).

## Results

Harman’s single-factor examination with EFA and CFA showed that CMV did not significantly impact our hypothesized variable relationships (Podsakoff et al., 2003). Specifically, in Harman’s single-factor EFA model, the unrotated first factor with the principle factor analysis explained 23.70% of the total variance. The model fit indices of Harman’s one-factor CFA model were poor. Yet, given that Harman’s single-factor examination is insensitive in examining CMV, we further examined and controlled for an unmeasured latent methods factor, with all the items loaded on this latent methods factor and trait factors they were assumed to measure (Podsakoff et al., 2003). A comparison of the latent methods factor model (*χ^2^* = 1028.57, d*f* = 582, *χ^2^*/d*f* = 1.77, RMSEA = 0.06, CFI = 0.92, NNFI = 0.91) and the theoretical model (*χ*^2^ = 1171.26, d*f* = 620, *χ*^2^/d*f* = 1.89, RMSEA = 0.06, CFI = 0.91, NNFI = 0.89) indicated a slight change of NNFI. Following Little (1997) advice, the NNFI change is less than 0.05, indicating that adding the latent methods factor did not significantly improve the theoretical model.

For CFA, based on the baseline model – a ten-factor model, we built twelve nested models. As shown in **Table 1**, results indicated that the ten-factor model had better model fit indices than all other nested models. Thus, the ten variables of interest in this study had sufficient discriminant validity. Comparing Harman’s one-factor CFA model with the baseline ten-factor CFA model, there was a significance change of *χ^2^* value [*χ^2^*(6) = 4091.70, *p* < 0.001]. These results further indicate that CMV was not a concern in this study and did not have an essential impact on our hypothesized relationships.

**Table 1:** Results of confirmatory factor analysis.

Model	*χ^2^*	d*f*	*χ*^2^/d*f*	RMSEA	CFI	TLI	Δ*χ^2^*	Δ*df*
Ten-factor model	1171.26	620	1.89	0.061	0.91	0.89		
Nine-factor model 1	1381.76	629	2.20	0.071	0.87	0.86	210.50^∗∗∗^	9
Nine-factor model 2	1531.31	629	2.43	0.078	0.84	0.83	360.05^∗∗∗^	9
Nine-factor model 3	1582.69	629	2.52	0.08	0.84	0.82	411.43^∗∗∗^	9
Nine-factor model 4	1617.40	629	2.57	0.08	0.83	0.81	446.14^∗∗∗^	9
Nine-factor model 5	1471.98	629	2.34	0.075	0.85	0.84	300.72^∗∗∗^	9
Eight-factor model 1	1714.89	637	2.69	0.086	0.81	0.79	543.63^∗∗∗^	17
Eight-factor model 2	1876.70	637	2.95	0.091	0.79	0.76	705.44^∗∗∗^	17
Five-factor model	2816.53	655	4.30	0.118	0.63	0.60	1645.27^∗∗∗^	35
Four-factor model	3069.65	659	4.66	0.124	0.58	0.56	1898.39^∗∗∗^	39
Three-factor model	3810.06	662	5.76	0.142	0.46	0.42	2638.80^∗∗∗^	42
Two-factor model	4488.46	664	6.76	0.156	0.34	0.30	3317.20^∗∗∗^	44
One-factor model	4776.02	665	7.18	0.162	0.29	0.25	3604.76^∗∗∗^	9

*N = 236, ^∗^p < 0.05, ^∗∗^p < 0.01,^∗∗∗^p < 0.001. Ten-factor model: wife’s family involvement, husband’s family involvement, wife’s work-family enhancement, husband’s work-family enhancement, wife’s positive affect, husband’s positive affect, wife’s family-work conflict, husband’s family-work conflict, wife’s job satisfaction, and husband’s job satisfaction. Nine-factor model 1: wife’s family involvement + husband’s family involvement, wife’s work-family enhancement, husband’s work-family enhancement, wife’s positive affect, husband’s positive affect, wife’s family-work conflict, husband’s family-work conflict, wife’s job satisfaction, and husband’s job satisfaction. Nine-factor model 2: wife’s family involvement, husband’s family involvement, wife’s work-family enhancement + husband’s work-family enhancement, wife’s positive affect, husband’s positive affect, wife’s family-work conflict, husband’s family-work conflict, wife’s job satisfaction, and husband’s job satisfaction. Nine-factor model 3: wife’s family involvement, husband’s family involvement, wife’s work-family enhancement, husband’s work-family enhancement, wife’s positive affect + husband’s positive affect, wife’s family-work conflict, husband’s family-work conflict, wife’s job satisfaction, and husband’s job satisfaction. Nine-factor model 4: wife’s family involvement, husband’s family involvement, wife’s work-family enhancement, husband’s work-family enhancement, wife’s positive affect, husband’s positive affect, wife’s family-work conflict + husband’s family-work conflict, wife’s job satisfaction, and husband’s job satisfaction. Nine-factor model 5: wife’s family involvement, husband’s family involvement, wife’s work-family enhancement, husband’s work-family enhancement, wife’s positive affect, husband’s positive affect, wife’s family-work conflict, husband’s family-work conflict, wife’s job satisfaction + husband’s job satisfaction. Eight-factor model 1: wife’s family involvement + husband’s family involvement + wife’s work-family enhancement + husband’s work-family enhancement, wife’s positive affect, husband’s positive affect, wife’s family-work conflict, husband’s family-work conflict, wife’s job satisfaction, and husband’s job satisfaction. Eight-factor model 2: wife’s family involvement, husband’s family involvement, wife’s work-family enhancement, husband’s work-family enhancement, wife’s positive affect + husband’s positive affect, wife’s family-work conflict, husband’s family-work conflict, wife’s job satisfaction + husband’s job satisfaction. Five-factor model: wife’s family involvement + husband’s family involvement, wife’s work-family enhancement + husband’s work-family enhancement, wife’s positive affect + husband’s positive affect, wife’s family-work conflict + husband’s family-work conflict, wife’s job satisfaction + husband’s job satisfaction. Four-factor model: wife’s family involvement + husband’s family involvement, wife’s work-family enhancement + husband’s work-family enhancement, wife’s positive affect + husband’s positive affect + wife’s job satisfaction + husband’s job satisfaction, wife’s family-work conflict, husband’s family-work conflict. Three-factor model: wife’s family involvement + husband’s family involvement + wife’s work-family enhancement + husband’s work-family enhancement, wife’s positive affect + husband’s positive affect + wife’s job satisfaction + husband’s job satisfaction, wife’s family-work conflict + husband’s family-work conflict. Two-factor model: wife’s family involvement + wife’s work-family enhancement + wife’s positive affect + wife’s family-work conflict + wife’s job satisfaction, husband’s family involvement + husband’s work-family enhancement + husband’s positive affect + husband’s job satisfaction + husband’s family-work conflict. One-factor model: wife’s family involvement + wife’s work-family enhancement + wife’s positive affect + wife’s family-work conflict + wife’s job satisfaction + husband’s family involvement + husband’s work-family enhancement + husband’s positive affect + husband’s job satisfaction + husband’s family-work conflict.*

**Table 2** shows the means, standard deviations, and correlations among variables. To justify the aggregation of the three variables – work-to-family enhancement, positive affect, and family-work conflict, we report *R_wg_*, ICC1, and ICC2. The *R_wg_* values were all above 0.70, showing strong within-dyad consistency (George and Bettenhausen, 1990). The ICC1 of the three variables was 0.44, 0.36, and 0.36, respectively, all of which were above 0.12 (James, 1982). ICC2 of the three variables was 0.61, 0.53, and 0.53, respectively, all of which were above 0.50 (James, 1982).

**Table 2:** Descriptive statistics and correlation for variables at the individual level.

Variable	Mean	*SD*	1	2	3	4	5	6	7	8	9	10	11	12	13	14	15	16	17	18	19
1 Age (wife)	38.63	6.61								
2 Tenure (wife)	12.50	8.61	0.66^∗∗∗^							
3 Work time (wife)	45.67	11.63	−0.05	0.02						
4 Family time (wife)	15.90	11.87	−0.11	−0.15^∗^	0.09					
5 Age (husband)	40.82	6.93	0.94^∗∗∗^	0.65^∗∗∗^	−0.03	−0.15				
6 Tenure (wife)	14.67	9.41	0.63^∗∗∗^	0.55^∗∗∗^	−0.07	−0.14^∗^	0.65^∗∗∗^			
7 Work time (husband)	46.81	15.53	−0.03	−0.06	0.32^∗∗∗^	0.11	−0.04	−0.12		
8 Family time (husband)	13.61	11.83	−0.19^∗∗^	−0.11	0.06	0.44^∗∗∗^	−0.21^∗∗^	−0.10	0.04	
9 Number of children	0.86	0.35	0.32^∗∗∗^	0.15^∗^	0.01	0.07	0.31^∗∗∗^	0.22^∗∗^	−0.03	−0.10
10 Family involvement (wife)	4.12	0.98	−0.24^∗∗∗^	−0.13^∗^	0.07	0.10	−0.23^∗∗∗^	−0.20^∗∗^	0.00	0.02	0.10	(0.87)									
11 Work-to-family enhancement (wife)	4.15	1.00	0.06	−0.00	−0.08	0.04	0.06	0.01	−0.00	−0.13	−0.11	−0.06	(0.87)								
12 Positive affect (wife)	4.36	0.78	−0.02	−0.04	−0.07	0.03	−0.01	0.02	−0.01	−0.03	0.02	−0.03	0.39^∗∗∗^	(0.86)							
13 Family-work conflict (wife)	1.93	0.88	−0.10	0.02	−0.05	0.15^∗^	−0.13	−0.09	−0.05	0.04	0.08	0.22^∗∗^	−0.26^∗∗∗^	−0.21^∗∗^	(0.89)						
14 Job satisfaction (wife)	4.25	0.88	−0.01	−0.08	0.00	−0.09	0.01	−0.03	−0.08	−0.07	−0.01	−0.21^∗∗^	0.56^∗∗∗^	0.39^∗∗∗^	−0.31^∗∗∗^	(0.83)					
15 Family involvement (husband)	4.02	0.97	−0.18^∗∗∗^	−0.11	0.15^∗^	0.09	−0.19^∗∗^	−0.19^∗∗^	−0.00	0.21^∗∗^	0.01	0.56^∗∗∗^	−0.05	−0.05	0.08	−0.14^∗^	(0.86)				
16 Work-to-family enhancement (husband)	4.19	0.97	0.04	0.04	0.02	0.01	0.03	−0.02	−0.04	0.03	0.04	−0.04	0.44^∗∗∗^	0.31^∗∗∗^	−0.28^∗∗∗^	0.28^∗∗∗^	−0.00	(0.86)			
17 Positive affect (husband)	4.45	0.78	−0.03	0.04	0.07	−0.02	−0.03	−0.04	−0.08	0.04	0.07	−0.02	0.22^∗∗^	0.36^∗∗∗^	−0.10	0.24^∗∗∗^	0.06	0.42^∗∗∗^	(0.87)		
18 Family-work conflict (husband)	2.18	0.99	−0.09	0.00	−0.08	−0.03	−0.10	−0.06	−0.04	0.15^∗^	0.02	0.14^∗^	−0.30^∗∗∗^	−0.42^∗∗∗^	0.50^∗∗∗^	−0.25^∗∗∗^	0.11	−0.24^∗∗∗^	−0.16^∗^	(0.93)	
19 Job satisfaction (husband)	4.24	0.92	0.00	0.01	−0.01	−0.00	−0.02	−0.03	−0.06	0.13	0.05	−0.13^∗^	0.23^∗∗∗^	0.24^∗∗∗^	−0.21^∗∗^	0.36^∗∗∗^	−0.10	0.49^∗∗∗^	0.43^∗∗∗^	−0.19^∗∗^	(0.82)

*N = 236, ^∗^p < 0.05, ^∗∗^p < 0.01, ^∗∗∗^p < 0.001.*

Results of cross-level polynomial regressions are reported in **Table 3**. In Model 1, job satisfaction was regressed on a set of control variables, while in Model 2 it was regressed on the controls and the five polynomial terms of family involvement (FI) (husband FI, wife FI, husband FI ^∗^ husband FI, wife FI ^∗^ wife FI, and husband FI^∗^ wife FI) (hereafter, the five polynomial terms refer to these five terms). Comparing Model 1 with 2, there was a significant change in *R*^2^ (Δ*R*^2^ = 0.04, *p* < 0.01), indicating that the five polynomial terms of family involvement were all jointly significant; meanwhile, the slope of the congruence line (Family involvement = Spouse family involvement or FI = SFI) was significantly negative (*β* = −0.20, *p* < 0.001), indicating that individual job satisfaction was lower for high-high spouses’ family involvement than for low-low family involvement. Thus, hypothesis 1 was supported.

**Table 3:** Results of cross-level polynomial regressions.

Variable	Job satisfaction			Positive affect		
	Model 1	Model 2	Model 3	Model 4	Model 5	Model 6
Age	0.01	0.01	0.00	0.01	0.00	0.00
Number of children	0.09	0.17	0.22	0.17	0.19	0.27^∗^
Tenure	−0.01	−0.01	−0.01	−0.01	−0.01	−0.01
Work time	−0.00	−0.00	−0.00	−0.00	−0.00	−0.00
Family time	0.00	0.00	0.00	0.00	0.00	0.00
FI		−0.14^∗∗^		0.03	0.04	0.06
SFI		−0.06		−0.05	−0.05	−0.04
FI^2^		0.01		0.06	0.06	0.07
FI ^∗^ SFI		−0.04		0.09	0.09	0.07
SFI^2^		0.06		0.03	0.03	0.04
WFE					0.35^∗∗∗^	0.48^∗∗∗^
WFE ^∗^ FI						−0.05
WFE ^∗^SFI						−0.03
WFE ^∗^ FI^2^						−0.03
WFE ^∗^ FI^∗^SFI						0.07
WFE ^∗^SFI^2^						−0.12
*R^2^*	0.01	0.05^∗^	0.01	0.06^∗∗^	0.20^∗∗∗^	0.39^∗∗∗∗^
Δ*R^2^*		0.04^∗∗^		0.04^∗∗^		0.19^∗∗∗^
Congruence line (FI = SFI)						
Slope		−0.20^∗∗∗^		−0.02		0.02
Curvature		0.03		0.17^∗∗∗^		0.18^∗∗∗^
Incongruence line (FI = −SFI)						
Slope		−0.08		0.08		0.11
Curvature		0.10		−0.01		0.04

*N = 236, ^∗^p < 0.05, ^∗∗^p < 0.01, ^∗∗∗^p < 0.001. FI, SFI, and WFE, respectively, stands for family involvement, spouse family involvement, and work-to-family enhancement.*

Model 3 and 4 were used to test hypotheses 2a and 2b. In Model 3, positive affect was regressed on a set of control variables, while in Model 4 it was regressed on the controls and the five polynomial terms. Results showed that comparing Model 4 with Model 3, R^2^ had a significant change (Δ*R*^2^ = 0.04, *p* < 0.01), indicating that the five polynomial terms were jointly significant. Although the slope was not significantly different from zero (*β* = −0.02, *p* > 0.05), the curvature along the congruence line (FI = SFI) was significantly positive (*β* = 0.17, *p* < 0.001). These results implied the congruence effect of spouses’ family involvement on positive affect at the family level. The surface response plot showed that the surface was curved upward and essentially flat at the point of incongruence (based on the insignificant slope along the FI = SFI line). That is, high-high spouses’ family involvement was positively related to positive affect at the family level, while low-low spouses’ family involvement was negatively related to positive affect at the family level. Thus hypothesis 2a was supported. However, because the slope (*β* = 0.08, *p* > 0.05) and the curvature (*β* = −0.01, *p* > 0.05) along the incongruence line (FI = −SFI) were both insignificant, it suggests that the incongruence of spouses’ family involvement was not significantly associated with positive affect at the family level, and thus hypothesis 2b was not supported.

**FIGURE 2:**
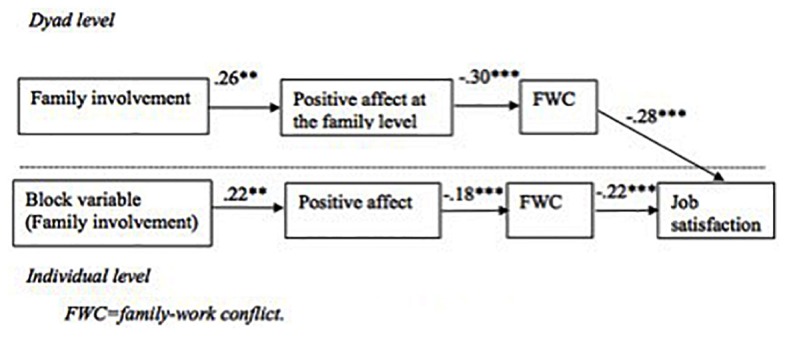
Result of cross-level SEM with a block variable. ^∗∗∗^*p* < 0.001.

The hypothesized theoretical model in **Figure 2** is used to test hypothesis 3a. In *Mplus*, we used the model constraint command to estimate the mediating effect (or indirect effect, a^∗^b). This command calculates the standard error (SE) of the mediating effect with the Delta method and further calculates the *t* value and the *p-*value of mediation. The formula is as follows: SE_ab_ = $\sqrt{a^{2}{\mathrm{se}}_{\text{b}}^{2}+b^{2}{\mathrm{se}}_{\text{a}}^{2}+{\mathrm{se}}_{\text{a}}^{2}{\mathrm{se}}_{\text{b}}^{2}}$, where *a* is the regression coefficient of the independent variable on the mediator, *b* is the regression coefficient of the mediator on dependent variable, *se_a_* is the standard error of *a*, and *se_b_* is the standard error of *b*^1^. Positive affect at the family level had a significant negative impact on family-work conflict (*β* = −0.30, *p* < 0.001), while family-work conflict had a significant negative impact on individual job satisfaction (*β* = −0.28, *p* < 0.001), and the mediating effect (−0.30 ^∗^ −0.28) was significant (*β* = 0.08, *p* < 0.01). The results indicated that family-work conflict mediated the relationship between positive affect at the family level and individual job satisfaction. Thus hypothesis 3 was supported. Family involvement had a significant positive impact on positive affect at the family level (*β* = 0.26, *p* < 0.001), and the double mediating effect (0.26 ^∗^ −0.30 ^∗^ −0.28) of positive affect the family level and family-work conflict on family involvement and job satisfaction was significant (*β* = 0.02, *p* < 0.05). The results indicated that positive affect at the family level and family-work conflict mediated the relationship between family involvement and job satisfaction. Thus hypothesis 3a was supported.

Model 5 and 6 in **Table 3** were used to test the moderating effect of working couple’s perceived work-to-family enhancement. Comparing Model 6 with 5, *R^2^* had a significant change (Δ*R^2^* = 0.19, *p* < 0.001), indicating that the product terms between the working couple’s perceived work-to-family enhancement and the five polynomial terms were jointly significant. Although the slope was not significantly different from zero (*β* = 0.02, *p* > 0.05), the curvature (*β* = 0.18, *p* < 0.001) along the congruence line (FI = SFI) was significant. Thus working couple’s perceived work-to-family enhancement moderated the relationship between spouses’ family involvement and positive affect at the family level. However, since the slope (*β* = 0.11, *p* > 0.05) and curvature (*β* = 0.04, *p* > 0.05) along the incongruence line (FI = −SFI) were both insignificant, the incongruence effect was not supported and the overall moderating effect of working couple’s perceived work-to-family enhancement may not be supported in this situation.

**Table 4** shows the polynomial regression of the high and low working couple’s perceived work-to-family enhancement groups. For the low working couple’s perceived work-to-family enhancement group, the slope was not significant (*β* = 0.00, *p* > 0.05) but the curvature along the congruence line (FI = SFI) was positively significant (*β* = 0.40, *p* < 0.001). For the high working couple’s perceived work-to-family enhancement group, the slope was not significant (*β* = 0.01, *p* > 0.05) and the curvature along the congruence line (FC = SFC) was insignificant (*β* = 0.15, *p* > 0.05). This indicated that for the low working couple’s perceived work-to-family enhancement group, high-high spouses’ family involvement had a less stronger positive impact on positive affect at the family level than the high working couple’s perceived work-to-family enhancement group, while low-low spouses’ family involvement had a stronger negative impact on positive affect at the family level than the high working couple’s perceived work-to-family enhancement group. Hence, we conclude that working couple’s perceived work-to-family enhancement has a positive moderating effect on high-high spouses’ family involvement on positive affect at the family level, and a negative moderating effect on low-low spouses’ family involvement on positive affect at the family level. Thus, Hypothesis 4a was supported.

**Table 4:** Moderated polynomial regression.

Variable	Work-to-family enhancement	
	Low score group	High score group
Age	−0.02	0.01
Number of children	0.37	0.24
Job tenure	0.01	0.02
Work time	−0.00	−0.01
Family time	−0.01	0.01
FI	0.09	−0.06
SFI	−0.09	0.08
FI^2^	0.25^∗^	0.01
FI ^∗^ SFI	−0.06	0.36^∗∗^
SFI^2^	0.22^∗^	−0.22^∗∗^
*R^2^*	0.26^∗∗^	0.39^∗∗∗^
Congruence line (FI = SFI)		
Slope	0.00	0.01
Curvature	0.40^∗∗∗^	0.15
Incongruence line (FI = −SFI)		
Slope	0.18	−0.14
Curvature	0.53	−0.58^∗^

*N = 236, ^∗^p < 0.05, ^∗∗^p < 0.01, ^∗∗∗^p < 0.001. FI and SFI, respectively, stands for family involvement and spouse family involvement.*

For the low working couple’s perceived work-to-family enhancement group, the slope along the incongruence line (FI = −SFI) was insignificant (*β* = 0.18, *p* > 0.10), and the curvature along the incongruence line (FI = −SFI) was insignificant (*β* = 0.53, *p* > 0.05), thus not providing evidence for the hypothesized incongruence effect. For the high working couple’s perceived work-to-family enhancement group, although the slope was insignificant (*β* = −0.14, *p* > 0.10), the curvature along the incongruence line (FI = −SFI) was significantly negative (*β* = −0.58, *p* < 0.05) and curved downward as the surface response plot showed. This provides evidence that the incongruence of spouses’ family involvement had a negative association with positive affect at the family level – the higher the level of incongruence, the lower the level of positive affect at the family level. Thus the low level of working couple’s perceived work-to-family enhancement did not moderate the incongruence of family involvement and positive affect at the family level, but the high level of working couple’s perceived work-to-family enhancement moderated this relationship. Thus hypothesis 4b was partially supported.

## Discussion

This study builds on the resource accumulation perspective in the expansion model of personal resources (Frone, 2003; Greenhaus and Powell, 2006) to examine how various dynamics of working spouses’ family involvement impacts their job satisfaction. We find that family involvement at the whole interface of working couples initiated mechanisms such as positive affect at the family level and family-work conflict mitigating the direct negative impact of spouses’ family involvement on job satisfaction. We also find that working couple’s perceived work-to-family enhancement moderated the relationship between the (in)congruence of spouses’ family involvement and positive affect at the family level.

### Theoretical Implications

The theoretical framework and analyses in this study enrich our understanding of work-family interface research in the following aspects. First, extant studies have looked at how an individual’s involvement in family life impacts his or her job performance and satisfaction (Aryee et al., 2005; Hanson et al., 2006; McNall et al., 2010), but seldom examined the whole interface of working couples in one theoretical framework, especially examining the dynamic interaction between them in the family and/or work domain. In this regard, our analysis of the working couples’ family involvement advances extant research on family involvement at the individual level. We argue that at the dyad level, the couples’ family involvement and job satisfaction entails a non-linear complex relationship. As such, this study challenges and complements the extant viewpoint on the negative relationship between family involvement and job satisfaction (Hanson et al., 2006). Our study implies that when examining working couples, we need to differentiate different levels of dynamic interaction between the dyads as studies on leader and follower dyads do (Zhang et al., 2012).

Second, this study extends our understanding of family-to-work spillover effects by including positive affect at the family level, which is more likely to be created when both spouses are highly involved in family life. Extant research states that for an individual the positive spillover from family to work mainly derives from family support (Crouter, 1984; Michel et al., 2011), yet our research introduces another form of positive spillover deriving from family involvement, that is, positive affect at the family level for the working couple. Our findings show that high-high spouses’ family involvement decreased family-work conflict and indirectly increased their job satisfaction. Thus, an examination of positive affect at the family level not only extends extant research of positive spillover from family to work at the individual level (Rothbard, 2001) to the family level, but also serves as a mechanism of how spouses’ family involvement impacts job satisfaction.

Third, we examined spouses’ work-to-family spillover from the marital dyad perspective (i.e., family level) using working couple’s perceived work-to-family enhancement as a moderator, which has rarely been examined in previous research (e.g., Aryee et al., 2005; Hanson et al., 2006). In the present study, we uncover how collective effects between the working couples from the work (working couple’s perceived work-to-family enhancement) affect their family life. Our findings indicate that working couples’ work-to-family enhancement positively moderated the positive relationship between high-high spouses’ family involvement and positive affect at the family level, and negatively moderated the negative relationship between low-low spouses’ family involvement and positive affect at the family level. Yet only the high level of working couples’ work-to-family enhancement moderated the negative relationship between the incongruence of spouses’ family involvement and positive affect at the family level. These findings demonstrate how the relationship between the dynamic interactions of spouses’ family involvement and positive affect at the family level differs at different levels of working couple’s perceived work-to-family enhancement.

Fourth, the present study undertakes an exploration of work-family interface across levels (i.e., individual and family), which is appropriate to demonstrate the dynamic process of work-family interface. The work-family interface is not just about an individual but a concern of the family especially the working couples. Hence our study contributes to the call to study work-family interface from a cross-level perspective involving individual and family levels (Frone, 2003; Eby et al., 2005).

### Practical Implications

Our findings suggest that high-high spouses’ family involvement may have an indirect positive impact on job satisfaction by stimulating positive affect at the family level and mitigating family-work conflict. Integrating this viewpoint into human resource management, managers are suggested to break the rigid work-life boundaries of employees and reduce employee’s job stressors (e.g., Morris, 2008). For instance, managers may allow employees to have more flexibility at work, which gives them time and energy to take care of families. Organizations may also organize various entertainment activities and invite families of employees to spend some fun time together. These strategies will provide spouses with a bigger opportunity to create a positive affect at the family level and promote their respective job satisfaction.

In addition, our findings have practical implications to teachers regarding their work-life balance. A high level of work-to-family enhancement increases the positive relationship between spouses’ family involvement and positive affect at the family level. Schools may create a culture of teacher appreciation (e.g., set up teach appreciate day each week) to increase teacher’s felt value at work, which enriches teachers’ family life.

### Limitations and Future Directions

One limitation of this study is that the sample was collected from the primary school teachers and their spouses – working couples. Although there is a potential limitation of the generalizability of our research findings, previous studies have pointed out that work-life interference issues are more significant among primary teacher families (Yang et al., 2009). Another limitation is about the cross-sectional design of this study. To mitigate common method bias, we informed the husband and the wife to complete the survey separately to reduce their impact on each other. We also conducted empirical tests of CMV to mitigate its impact on our hypothesized relationships.

This study also suggests several future research directions. First, we suggest a longitudinal design to examine the relationship and mechanisms between spouses’ family involvement and job satisfaction. Second, given that children are an integrative component of family life in Chinese culture and the number of children has been mostly controlled in extant studies (e.g., Aryee et al., 2005; Hanson et al., 2006), for future research the inclusion of both marital dyads and children dyads would help us to better understand work-family dynamics. Second, given that in a cross-level study (i.e., individual and family levels) on work-family interface, the variable relationships are more likely to be impacted by family characteristics and workplace organizational characteristics such as structural work-life support in forms of policies, practices and job structures and cultural work-life support (Kossek et al., 2010), an exploration of these variables may be another direction in the future. Further, although in most cultures the wife and the husband share an equal position in the family domain, they may play different family roles (e.g., one focuses on making money, while the other focuses on family affairs), which probably could lead to job and family satisfactory at the same time. As such, a comparative study of samples with different cultural backgrounds would also be interesting and meaningful.

## Data Availability

The raw data supporting the conclusions of this manuscript will be made available by the authors, without undue reservation, to any qualified researcher.

## Ethics Statement

In this study, data were collected by HL at Beijing Normal University. The protocol was approved by the Beijing Social Science Foundation and funded as an important research project. Institutions in China do not have Institutional Review Board. As a protection of all participants, all subjects read informed consent before participating this study and voluntarily made their decision to complete surveys.

## Author Contributions

HW developed the theoretical model and hypotheses, and wrote the paper. JZ conducted all the data analyses and wrote the analysis report. HL collected the data. DB provided comments on different versions of the paper and edited the manuscript.

## Conflict of Interest Statement

The authors declare that the research was conducted in the absence of any commercial or financial relationships that could be construed as a potential conflict of interest.

## Footnotes

1 http://www.statmodel.com/discussion/messages/11/570.html?1509138764
